# Birth weight, childhood body mass index, and height in relation to mammographic density and breast cancer: a register-based cohort study

**DOI:** 10.1186/bcr3596

**Published:** 2014-01-20

**Authors:** Zorana J Andersen, Jennifer L Baker, Kristine Bihrmann, Ilse Vejborg, Thorkild IA Sørensen, Elsebeth Lynge

**Affiliations:** 1 Center for Epidemiology and Screening, Department of Public Health, University of Copenhagen, Øster Farimagsgade 5, 1014 Copenhagen, Denmark; 2 Institute of Preventive Medicine, Bispebjerg and Frederiksberg Hospital, The Capital Region, Frederiksberg Hospital, Hovedvejen 5, Nordre Fasanvej 57, 2000 Frederiksberg, Denmark; 3 Department of Large Animal Sciences, University of Copenhagen, Grønnegårdsvej 8, 1870 Frederiksberg C, Denmark; 4 Diagnostic Imaging Centre, Copenhagen University Hospital, Rigshospitalet, Blegdamsvej 9, 2100 Copenhagen, Denmark; 5 Novo Nordisk Foundation Centre for Basic Metabolic Research, Faculty of Health and Medical Sciences, University of Copenhagen, Blegdamsvej 3B, 2200 Copenhagen, Denmark

## Abstract

**Introduction:**

High breast density, a strong predictor of breast cancer may be determined early in life. Childhood anthropometric factors have been related to breast cancer and breast density, but rarely simultaneously. We examined whether mammographic density (MD) mediates an association of birth weight, childhood body mass index (BMI), and height with the risk of breast cancer.

**Methods:**

13,572 women (50 to 69 years) in the Copenhagen mammography screening program (1991 through 2001) with childhood anthropometric measurements in the Copenhagen School Health Records Register were followed for breast cancer until 2010. With logistic and Cox regression models, we investigated associations among birth weight, height, and BMI at ages 7 to 13 years with MD (mixed/dense or fatty) and breast cancer, respectively.

**Results:**

8,194 (60.4%) women had mixed/dense breasts, and 716 (5.3%) developed breast cancer. Childhood BMI was significantly inversely related to having mixed/dense breasts at all ages, with odds ratios (95% confidence intervals) ranging from 0.69 (0.66 to 0.72) at age 7 to 0.56 (0.53 to 0.58) at age 13, per one-unit increase in z-score. No statistically significant associations were detected between birth weight and MD, height and MD, or birth weight and breast cancer risk. BMI was inversely associated with breast cancer, with hazard ratios of 0.91 (0.83 to 0.99) at age 7 and 0.92 (0.84 to 1.00) at age 13, whereas height was positively associated with breast cancer risk (age 7, 1.06 (0.98 to 1.14) and age 13, 1.08 (1.00 to 1.16)). After additional adjustment for MD, associations of BMI with breast cancer diminished (age 7, 0.97 (0.88 to 1.06) and age 13, 1.01 (0.93 to 1.11)), but remained with height (age 7, 1.06 (0.99 to 1.15) and age 13, 1.09 (1.01 to 1.17)).

**Conclusions:**

Among women 50 years and older, childhood body fatness was inversely associated with the breast cancer risk, possibly via a mechanism mediated by MD, at least partially. Childhood tallness was positively associated with breast cancer risk, seemingly via a pathway independent of MD. Birth weight was not associated with MD or breast cancer in this age group.

## Introduction

Breast density, one of the strongest risk factors for breast cancer, may be determined prenatally or early in life [1,2]. A substantial number of studies have explored prenatal origins of breast cancer [3] by linking early-life anthropometric factors to breast cancer risk [3-6]. Birth weight, taken as a proxy for prenatal exposures [4,6-10], and childhood height [6,8,11,12] are both positively associated with breast cancer, whereas higher body mass index (BMI; kg/m^2^) [8,11,13-15] in childhood seems to protect against breast cancer. However, the period in life when breast density is determined, and whether it may mediate the effect of early-life exposures on breast cancer risk, is unknown [1,2].

Although few studies have linked early-life factors to mammographic density (MD) [16-25], they are limited by small numbers of subjects and self-reported anthropometric data [16,18-20,22-25], and, as such, yield inconsistent results. Studies on birth weight and MD offer mixed evidence, showing none [16-18] or significant and positive associations [19-21]. Studies on body size/BMI/weight/adiposity in childhood and MD show statistically significant and inverse associations in studies with predominantly Caucasian women [17,18,22-24], but none in Chinese immigrant women in the United States [25] or Mexican women [26]. It remains unclear whether an association exists between childhood height and MD, with three studies failing to detect a link [17,18,23], and a single study showing significant and positive associations [22]. One study that had data on childhood body size, MD, and breast cancer in the same population, concluded that MD did not explain the inverse association between childhood body fatness and premenopausal breast cancer [27].

Therefore, we linked measured data on body size at ages 7 to 13 years and records of birth weight, to records of MD and breast cancer after age 50 years, and explored whether MD mediates or modifies the association of birth weight, BMI, and height with breast cancer risk.

## Methods

### Study cohort

The study cohort consists of 13,572 women older than 50 years who participated in the Copenhagen mammography screening program between 1991 and 2001 and had childhood anthropometric data in the Copenhagen School Health Records Register (CSHRR).

### Childhood anthropometric data

The CSHRR is a database of health examinations records on 350,263 children born between 1930 and 1983 and who attended schools in Copenhagen municipality [28]. The health cards were filled out by school physicians or nurses who performed height and weight measurements on an annual basis from ages 5 through 7 until 13 through 17 (until 1984), whereas birth weight was reported by the parents who accompanied their child to the first visit (since 1936). The unique personal identification (CPR) number, part of the Danish Civil Registration System [29], was retrieved for more than 88% of the study population. BMI *z*-scores were calculated based on children from 1955 through 1960, when the prevalence of overweight and obesity were low and stable, and were performed by using the LMS (lambda-mu-sigma) method [30]. Height *z*-scores were calculated by using the LMS method and based on cohort-specific values, as increases in height occurred from the 1930 to 1983 birth years.

### MD definition

The Copenhagen mammography screening program started in 1991 [31] and targeted about 40,000 women aged 50 to 69 years at the start of each biennial invitation round. Women were free to refuse to participate in screening as well as to decline further invitations. We used data from the first screening for 134,640 women who participated in first five rounds of screening between 1991 and 2001 [32]. One radiologist was in charge of the screening, which occurred at a single Copenhagen hospital. Attending women were asked to fill in a questionnaire on hormone-replacement therapy (HRT) use, earlier breast surgery, family history of BC, and eventual suspicion of a breast lump. All screens were taken by the radiographers or x-ray nurses, and were evaluated independently by two radiologists, who did not meet the attending women, but knew their ages and the answers to the questionnaire, which, however, were not entered in a database, and were not available for this study. Age and birth-cohort information was available from each woman’s CPR number, as it contains the date of birth [29].

Two views were taken on the first screen, a craniocaudal and an oblique. MD was dichotomized into fatty breast, equivalent to Breast Imaging Reporting and Data System (BI-RADS; Atlas, 2008) density code 1 and part of code 2, and mixed/dense breast, equivalent to part of BI-RADS code 2, 3, or 4. Women with a negative screening test and fatty breasts were scheduled to have only an oblique view at the next screen, whereas women with a negative screening test and mixed/dense breasts were scheduled for two views. The dichotomous outcome for MD was successfully used earlier, showing expected associations with breast cancer risk [32].

Women were divided into four birth-year intervals (1930 to 1934, 1935 to 1939, 1940 to 1944, and 1945 to 1949) to account for birth-cohort effects. By using the CPR number [29], we linked the Copenhagen mammography register to the CSHRR, and identified 13,958 women with data in both.

### Breast cancer definition

We linked the records of 13,958 women by using the CPR number to the Danish Cancer Registry [33] to extract breast cancer diagnoses, including invasive and *in situ* cancers (ICD-10 codes C50 and D05) between screening (1991 to 2001) and 31 December 2009, and to the Civil Registration System [29] to extract information on emigration or death. We identified 1,087 cases of breast cancer, of which 288 were diagnosed before the screening date and excluded. We furthermore excluded 98 women without MD information, due to detection of breast cancer at screening, leaving 13,572 in main analyses.

### Statistical methods

We used logistic regression to investigate association of MD with birth weight, height, and BMI in separate models, in two steps: crude model (model 1) and in a model adjusted for birth cohort and age at the time of screening (model 2). We used Cox proportional hazards regression with age as the underlying time, to investigate associations of birth weight, height, and BMI, separately, with the risk of breast cancer, in three steps: a crude model (age adjusted as age underlies time scale) (model 1), a model 1 additionally stratified by birth cohort (model 2), and a model 2 additionally adjusted for MD (model 3). The follow-up started on the date of screening (1991 through 2001) until a date of breast cancer diagnosis, death, emigration, or December 31, 2009, whichever came first.

Comparison of hazard ratios (HRs) estimating the effect of birth weight, BMI, and height on the risk of breast cancer from model 2 (without MD) and model 3 (with MD) was made for evaluation of a possible mediating role of MD. Birth weight, BMI, and height *z*-scores at ages 7 through 13 were modeled as continuous variables, and a separate model was fit at each age.

Main analyses were performed in Stata 11.2. We additionally fit generalized additive models for binary/survival data with natural splines for birth weight, height, and BMI *z*-scores (*mgcv* and *design* packages, R statistical software 2.13.0), and evaluated the shape of associations with MD/breast cancer, both visually and by log-likelihood tests against the corresponding linear model. The potential effect modification of an association between birth weight, BMI, and height and breast cancer by age and MD was evaluated by introducing interaction terms into the Cox model, and tested by the Wald test.

A sensitivity analysis was performed on an extended population, including breast cancer cases among 98 women who did not have data on MD. Additional sensitivity analyses were performed excluding women diagnosed with breast cancer within 1 and 2 years of their mammogram, to address the possibility of masking bias driving the association between MD and breast cancer risk. Finally, a sensitivity analyses was performed on the associations of BMI and height with MD and breast cancer, respectively, excluding women with missing data on birth weight.

The study was entirely based on a data from Danish health registers and approved by the Danish Data Inspection Agency by Danish law serving as ethical approval of register-based research, which does not require an informed consent from study participants. Thus, no contact has been made with participating women, relatives, or their practicing doctors, and no consent was needed.

## Results

**Figure 1:**
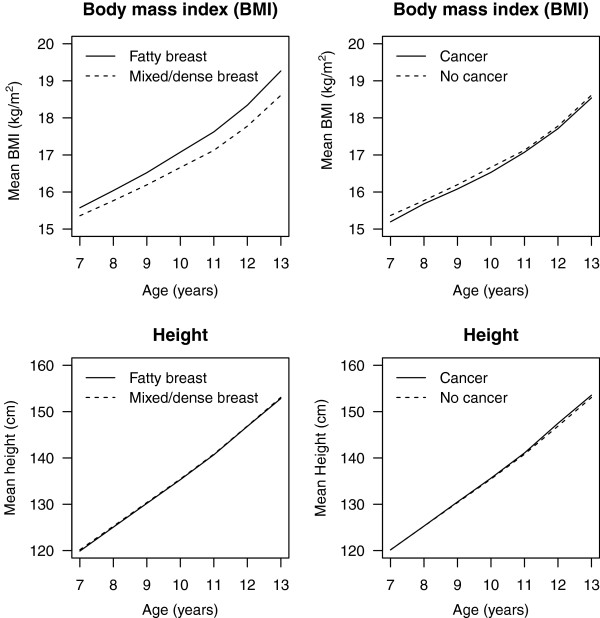
**Mean BMI (top) and height (bottom) at ages 7 to 13 years in 13,572 women, separately for women with fatty breasts (*n*** **= 5,378) and mixed/dense breasts (*n*** **= 8,194) (left), and in women free of breast cancer (*n*** **= 12,959) and with breast cancer (*n*** **= 613) (right); data from the Copenhagen School Health Records Register.**

The majority (60.4%) of women had mixed/dense breasts at a mean age of 54.6 years. Mean birth weight and BMI at all (7 through 13 years) ages was lower in women with mixed/dense breasts than in those with fatty breast, whereas no differences in height were found (Table 1, Figure 1). In total, 716 (5.3%) cases of breast cancer were diagnosed during 184,175 person-years of follow-up, with an incidence rate of 3.9 cases per 1,000 person-years. The mean age at diagnosis was 63 years, with the majority (92.2%) of cancers detected at older than age 55.

**Table 1:** Distribution of demographic, mammographic screening, and childhood anthropometric data for 13,572 women

	**Number of subjects**	**Total *N*** **= 13,572**	**Mammographic density**		**Breast cancer**	
			**Mixed/dense breasts *n*** **= 8,194**	**Fatty breasts *n*** **= 5,378**	**Breast cancer cases*n*** **= 716**	**No breast cancer n = 12,856**
N (%) born 1930-1934		2,765 (20.4)	1,255 (15.3)	1,510 (28.1)	153 (21.4)	2,612 (20.3)
N (%) born 1935-1939		3,484 (25.7)	1,834 (22.4)	1,650 (30.7)	234 (32.7)	3,259 (25.3)
N (%) born 1940-1944		4,035 (29.7)	2,594 (31.7)	1,441 (26.8)	213 (29.7)	3,822 (29.7)
N (%) born 1945-1949		3,288 (24.2)	2,511 (30.6)	777 (14.4)	116 (16.2)	3,172 (24.7)
Mean (SD) age at screening (years)	13,572	54.6 (3.4)	54.1 (3.1)	55.3 (3.5)		
N (%) with fatty breast		5,378 (39.6)	0	5,378 (100)	175 (24.4)	5,203 (40.5)
N (%) with mixed/dense breast		8,194 (60.4)	8,194 (100)	0	541 (75.5)	7,653 (59.5)
Mean (SD) birth weight (g)	8.271	3,330 (543)	3,324 (538)	3,340 (554)	3,302 (554)	3,331 (543)
Mean (SD) BMI at age 7 (kg/m^2^)	12.640	15.4 (1.3)	15.2 (1.2)	15.6 (1.5)	15.2 (1.3)	15.4 (1.3)
Mean (SD) BMI at age 8 (kg/m^2^)	12.887	15.8 (1.5)	15.6 (1.4)	16.0 (1.6)	15.7 (1.3)	15.8 (1.5)
Mean (SD) BMI at age 9 (kg/m^2^)	12.968	16.2 (1.6)	16.0 (1.5)	16.5 (1.7)	16.1 (1.5)	16.2 (1.6)
Mean (SD) BMI at age 10 (kg/m^2^)	13.014	16.7 (1.8)	16.4 (1.6)	17.1 (2.0)	16.5 (1.7)	16.7 (1.8)
Mean (SD) BMI at age 11 (kg/m^2^)	13.045	17.1 (2.0)	16.8 (1.8)	17.6 (2.1)	17.0 (1.9)	17.1 (2.0)
Mean (SD) BMI at age 12 (kg/m^2^)	13.050	17.8 (2.1)	17.4 (1.9)	18.3 (2.3)	17.6 (2.0)	17.8 (2.1)
Mean (SD) BMI at age 13 (kg/m^2^)	13.002	18.6 (2.3)	18.2 (2.1)	19.3 (2.5)	18.5 (2.1)	18.6 (2.3)
Mean (SD) Height at age 7 (cm)	12.636	120.2 (5.4)	120.3 (5.4)	119.9 (5.4)	120.3 (5.4)	120.2 (5.4)
Mean (SD) Height at age 8 (cm)	12.882	125.3 (5.7)	125.4 (5.6)	125.1 (5.7)	125.4 (5.5)	125.3 (5.7)
Mean (SD) Height at age 9 (cm)	12.963	130.4 (5.9)	130.5 (5.9)	130.2 (5.9)	130.6 (5.7)	130.3 (5.9)
Mean (SD) Height at age 10 (cm)	13.011	135.4 (6.2)	135.5 (6.2)	135.3 (6.2)	135.7 (6.0)	135.4 (6.2)
Mean (SD) Height at age 11 (cm)	13.039	140.8 (6.7)	140.9 (6.7)	140.7 (6.7)	141.1 (6.6)	140.8 (6.7)
Mean (SD) Height at age 12 (cm)	13.044	146.9 (7.4)	146.9 (7.4)	146.8 (7.4)	147.4 (7.3)	146.9 (7.4)
Mean (SD) Height at age 13 (cm)	12.991	153.1 (7.4)	153.2 (7.4)	152.8 (7.4)	153.5 (7.2)	153.1 (7.4)

SD, standard deviation; BMI, body mass index.

A significant and inverse association between age and MD diminished after adjusting for birth cohort (Table 2). The youngest women had 3.92 times higher odds of having mixed/dense breast than the oldest. We found a weak, inverse association between birth weight and MD, and between height and MD. BMI was significantly and inversely related to having mixed/dense breast at all ages, with odds ratios (ORs) (95% confidence intervals) ranging from 0.69 (0.66 to 0.72) at age 7 to 0.56 (0.53 to 0.58) at age 13 per one-unit increase in *z*-score of BMI; this corresponds to an OR of 0.45 (0.40 to 0.51) comparing the highest to the lowest 25^th^ percentile of BMI distribution at age 13.

**Table 2:** Association between breast density (odds of having mixed/dense breasts) and exposure variables from the Copenhagen mammography register and the Copenhagen School Health Records Registry in 13,572 women

	**Model 1**	**Model 2**
	**Crude model OR (95% CI)**	**Adjusted**^**a **^**model OR (95% CI)**
Age at screening (years)	0.90 (0.89-0.91)	1.00 (0.98-1.02)^b^
Born 1930 to 1934	1.00	1.00 ^c^
Born 1935 to 1939	1.34 (1.21-1.48)	1.34 (1.16-1.55)
Born 1940 to 1944	2.16 (1.96-2.39)	2.18 (1.80-2.65)
Born 1945 to 1949	3.89 (3.48-4.34)	3.92 (3.19-4.81)
Birth weight (g)	0.95 (0.87-1.03)	0.98 (0.90-1.07)
BMI at age 7 (kg/m^2^)	0.72 (0.70-0.75)	0.69 (0.66-0.72)
BMI at age 8 (kg/m^2^)	0.70 (0.67-0.73)	0.65 (0.62-0.68)
BMI at age 9 (kg/m^2^)	0.67 (0.64-0.69)	0.62 (0.59-0.64)
BMI at age 10 (kg/m^2^)	0.64 (0.61-0.67)	0.59 (0.57-0.62)
BMI at age 11 (kg/m^2^)	0.61 (0.59-0.64)	0.57 (0.55-0.60)
BMI at age 12 (kg/m^2^)	0.60 (0.58-0.63)	0.56 (0.54-0.59)
BMI at age 13 (kg/m^2^)	0.59 (0.56-0.61)	0.56 (0.53-0.58)
Height at age 7 (cm)	0.99 (0.96-1.03)	0.99 (0.95-1.03)
Height at age 8 (cm)	0.99 (0.96-1.03)	0.98 (0.95-1.02)
Height at age 9 (cm)	0.99 (0.95-1.02)	0.98 (0.94-1.01)
Height at age 10 (cm)	0.97 (0.94-1.01)	0.96 (0.93-1.00)
Height at age 11 (cm)	0.96 (0.93-1.00)	0.95 (0.92-0.99)
Height at age 12 (cm)	0.96 (0.93-0.99)	0.95 (0.91-0.98)
Height at age 13 (cm)	0.98 (0.95-1.01)	0.96 (0.93-1.00)

OR, odds ratio; CI, confidence interval; BMI, body mass index. ^a^Adjusted for age at screening and birth cohort; ^b^adjusted for birth cohort; ^c^adjusted for age at screening.

**Figure 2:**
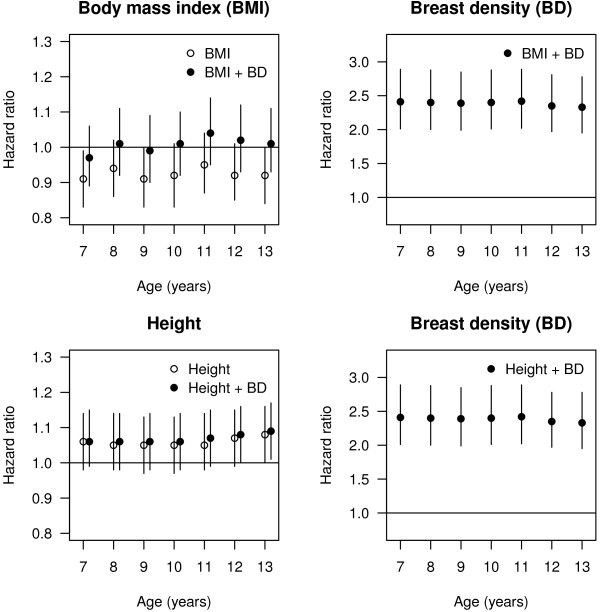
Association HRs and 95% confidence intervals (age underlying time scale, stratified by birth cohort) between breast cancer and BMI (top left) and height (bottom left), without (open circle) and with (solid circle) adjustment for breast density; and association (age underlying time scale, stratified by birth cohort) between breast cancer and breast density in a model adjusted for BMI (top right) and height (bottom right), in 13,572 women.

Having mixed/dense breasts more than doubled the breast cancer risk (hazard ratio (HR); 95% CI, 2.34; 1.97 to 2.78) compared with women with fatty breasts (Table 3). When excluding 82 and 123 women diagnosed with breast cancer within 1 and 2 years of their mammogram, respectively, this association remained unchanged, with HR of 2.32 (1.94 to 2.75), and 2.29 (1.91 to 2.73), respectively. Birth weight showed a weak, inverse association with breast cancer with HR of 0.89 (0.75 to 1.06), which remained unchanged after adjustment for MD. BMI was inversely associated with breast cancer at all ages, with statistically significant association at age 7, with an HR of 0.91 (0.83 to 0.99) per *z*-score unit, or 0.84 (0.67 to 1.05) when comparing the upper with the lower 25^th^ percentile of BMI distribution. After adjustment for MD, the association at age 7 was attenuated to 0.97 (0.88 to 1.06) and 0.97 (0.77 to 1.21), respectively, and became positive for all other ages (Table 3, Figure 2). Height was weakly and positively associated with breast cancer risk, with HRs ranging from 1.06 (0.98 to 1.14) at age 7 to 1.08 (1.00 to 1.16) at age 13, which remained after adjustment for MD.

**Table 3:** Association between breast cancer and exposure variables from Copenhagen mammography register and Copenhagen school health records registry in 13,572 women

	**Model 1**	**Model 2**	**Model 3**
	**Crude**^**a **^**model HR (95% CI)**	**Adjusted**^**b **^**model HR (95% CI)**	**Adjusted**^**c **^**model HR (95% CI)**
Fatty breast	1.00	1.00	-
Mixed/dense breast	2.26 (1.90-2.68)	2.34 (1.97-2.78)	-
Birth weight (g)	0.90 (0.75-1.07)^a^	0.89 (0.75-1.06)	0.88 (0.74-1.05)
BMI (kg/m^2^) age 7	0.91 (0.84-1.00)	0.91 (0.83-0.99)	0.97 (0.88-1.06)
BMI (kg/m^2^) age 8	0.94 (0.86-1.03)	0.94 (0.86-1.02)	1.01 (0.92-1.11)
BMI (kg/m^2^) age 9	0.91 (0.83-1.00)	0.91 (0.83-1.00)	0.99 (0.90-1.09)
BMI (kg/m^2^) age 10	0.92 (0.84-1.01)	0.92 (0.83-1.01)	1.01 (0.92-1.10)
BMI (kg/m^2^) age 11	0.95 (0.87-1.04)	0.95 (0.87-1.04)	1.05 (0.95-1.14)
BMI (kg/m^2^) age 12	0.93 (0.85-1.01)	0.92 (0.85-1.01)	1.02 (0.93-1.12)
BMI (kg/m^2^) age 13	0.92 (0.84-1.00)	0.92 (0.84-1.00)	1.01 (0.93-1.11)
Height (cm) age 7	1.06 (0.98-1.14)	1.06 (0.98-1.14)	1.06 (0.99-1.15)
Height (cm) age 8	1.05 (0.98-1.14)	1.05 (0.98-1.14)	1.06 (0.98-1.14)
Height (cm) age 9	1.05 (0.97-1.13)	1.05 (0.97-1.13)	1.06 (0.98-1.14)
Height (cm) age 10	1.05 (0.97-1.13)	1.05 (0.97-1.13)	1.06 (0.98-1.14)
Height (cm) age 11	1.05 (0.98-1.14)	1.05 (0.98-1.14)	1.07 (0.99-1.15)
Height (cm) age 12	1.07 (0.99-1.15)	1.07 (0.99-1.15)	1.08 (1.01-1.16)
Height (cm) age 13	1.08 (1.00-1.16)	1.08 (1.00-1.16)	1.09 (1.01-1.17)

CI, confidence interval; HR, hazard ratio; ^a^Adjusted for age (underlying time scale); ^b^Adjusted for age (underlying time scale) and stratified by birth cohort (1930 to 1934, 1935 to 1939, 1940 to 1944, 1945 to 1949). ^c^Adjusted for age (underlying time scale) and mammographic density, and stratified by birth cohort (1930 to 1934, 1935 to 1939, 1940 to 1944, 1945 to 1949).

No significant effect modifications were detected, expect for a borderline significant interaction (*P* value = 0.08) indicating that the positive association between height and breast cancer may be limited to women with mixed/dense breasts (Table 4).

**Table 4:** **Effect modification of an association**^**a**^**between breast cancer and exposure variables by age and mammographic density in 13,572 women**

	**Breast cancer *N *** **(%)**	**Birth weight HR (95% CI)**	**BMI at age 13 HR (95% CI)**	**Height at age 13 HR (95% CI)**
Age at BC diagnoses (years)				
50-59 years	193 (27.0)	1.01 (0.75-1.36)	1.03 (0.88-1.22)	1.06 (0.92-1.23)
60^+^ years	523 (73.0)	0.82 (0.66-1.02)	1.00 (0.90-1.11)	1.10 (1.00-1.20)
P value for interaction		0.31	0.81	0.69
Mammographic density				
Mixed/dense breasts	541 (75.5)	0.88 (0.72-1.07)	1.02 (0.92-1.14)	1.13 (1.03-1.23)
Fatty breasts	175 (24.4)	0.92 (0.62-1.36)	1.00 (0.81-1.17)	0.97 (0.83-1.13)
P value for interaction		0.85	0.70	0.08
Total	716	0.89 (0.75-1.06)	1.01 (0.93-1.11)	1.09 (1.01-1.17)

CI, confidence interval; HR, hazard ratio; ^a^Adjusted for age (underlying time scale) and mammographic density, and stratified by birth cohort (1930 to 1934, 1935 to 1939, 1940 to 1944, 1945 to 1949).

In a sensitivity analysis including 98 women with additional 75 confirmed cases of breast cancer, we found similar associations of BMI with breast cancer risk, as seen in main analyses (not shown). Finally, in analyses limited to women with available data on birth weight, we found similar associations of BMI and height with MD and breast cancer as in the main analyses with the maximum available data for each anthropometric measure (not shown).

## Discussion

High BMI in childhood was strongly inversely associated with developing mixed/dense breasts and marginally with breast cancer after age 50. MD may explain the inverse association between childhood BMI and breast cancer risk, at least in part. Tallness in childhood was not significantly associated with MD, but it was marginally positively associated with breast cancer risk. Birth weight was not significantly related to MD or breast cancer risk.

Inverse associations between BMI in childhood and MD in this cohort agree with existing evidence based primarily on Caucasian women in European and American populations [17,18,22-24], despite different definitions of MD and body size. The only study similar to ours with measured body size (ages 2 to 15 years) in 1,298 British women found an odds of a higher Wolf grade of 0.56 (0.49 to 0.64) per 2.8 kg/m^2^ in BMI at age 15 [17]; this is remarkably similar to ours of 0.56 (0.53 to 0.58) per *z*-score at age 13 years. A study of 628 Scottish women detected an inverse association between having a high-risk mammogram (≥ 25% dense) and BMI at age 18 [18]. Mammographic percentage density (MPD) in 1,893 American women was linked to self-reported weight and adiposity at 7, 12, and 18 years, but a significant inverse association was detected only at age 12 [22]. A significant inverse relation between MPD and self-reported weight before menarche was detected in 3,547 Spanish women [23]. Finally, self-reported BMI at age 8–10 years was significantly inversely associated with percentage and absolute breast density volume in 174 young women aged 25 to 30 years [24].

Our findings disagree with two studies failing to detect association between MD and self-reported weight at age 10 in 201 US Chinese immigrants [25] and self-reported body size before and after menarche in 1,531 Mexican women [26]. Overall evidence suggests possible relevance of race/ethnicity in the association of childhood body size and MD, with consistent and robust inverse associations observed in Caucasian women of European and American descent, and none in Asian or Hispanic women.

A weak protective effect of BMI at ages 7 through 13 on breast cancer risk in this cohort is confirmatory [8,11,13-15]. However, our finding that the inverse association between childhood BMI and breast cancer diminishes after adjustment for MD conflicts with Harris *et al*. [27], where this association was robust to adjustment for MD. Various differences between two studies preclude direct comparisons and possibly explain the conflicting results. Where we benefited from measured childhood anthropometrics and prospective cohort design, Harris *et al.*[27]*,* in a nested case–control study, retrospectively collected body fatness of women in Nurses’ Health Study at ages 5, 10, and 20 years, by using nine-level figure drawing. Conversely, although we adjusted for birth cohort and age in full model, lacking information on other breast cancer risk factors, Harris *et al.* matched cases and controls on age, menopausal status, postmenopausal hormone use, and race/ethnicity, and furthermore adjusted for age at menarche, parity/age at first birth, history of breast cancer, and alcohol use, but did not adjust for birth cohort.

Furthermore, breast cancer and MD definitions differ between the studies. Whereas we obtained mammograms and breast cancer information objectively via registries, without possibility for population selection by refusal to participate/release information, Harris *et al*. relied on self-reports confirmed by medical records in women who gave permission to obtain medical records and mammograms. Additionally, our study was conducted predominantly on postmenopausal women, whereas Harris *et al*. included a younger population of women at screening with a shorter follow-up (mean time between screening and breast cancer diagnoses of 4.7 years versus 8.6 years in our study), resulting in predominantly premenopausal breast cancer.

Finally, whereas we excluded 98 breast cancer cases diagnosed at screening from main analyses, because of lack of MD, Harris *et al*. allowed these in analyses [27]. In a sensitivity analysis including 98 women with additional 75 confirmed cases of breast cancer, we found similar associations of BMI with breast cancer risk as seen in main analyses (not shown).

The lack of an association between height at ages 7 to 13 and MD in our study concurs with two studies with measured heights at ages 2 to 15 [17] and at 18 [18], and a study with self-reported height before menarche [23]. A single study detected significant positive associations between self-reported height at ages 7, 12, and 18 with PMD [22]. The positive and significant associations between childhood height and breast cancer risk in this cohort corroborate current evidence [6,8,11,12], but none of the previous studies tested whether this association could be explained by MD. Likewise, our finding of a borderline significant interaction (*P* value = 0.08), indicating that the positive association between height and breast cancer may be limited to women with mixed/dense breasts (Table 4), calls for replication.

Birth weight was not associated with MD in this cohort, consistent with three [16-18] and in contrast to three studies showing significant positive associations [19-21]. Cerhan *et al*. found significant positive associations of birth weight with MPD only in the postmenopausal group of the 1,893 US women [19], whereas Tamimi *et al*. [20] presented data on 893 Swedish postmenopausal women only. Pearce e*t* a*l*. [21] detected significant and positive associations in a mixed group of 199 pre- and postmenopausal British women. The differences between our and these studies [19-21] may be due to adjustment for additional covariates, such as BMI/weight at mammography, HRT use, menopausal status, parity, age at first pregnancy, alcohol consumption, and so on. Pearce *et al*. [17-20] showed that adjustment for the most complete set of confounders of all mentioned studies resulted in higher and statistically significant effect estimates of an association between birth weight and MD, as compared with a crude estimate [21]. However, studies failing to detect an association between birth weight and MD did not observe differences between crude and adjusted models [17,18]. Additional adjustment for BMI at age 13 in our analyses, next to birth cohort and age at screening, changed our OR from 0.98 (0.90 to 1.07) to 1.11 (1.02 to 1.22), in agreement with Pearce *et al*. [21]. However, we chose not to adjust for BMI at age 13 in main analyses, as childhood BMI may be an intermediate variable on the causal pathway between birth weight and MD. In any case, a critical assessment of relevant covariate adjustment is necessary when comparing estimates of association between birth weight and MD.

We found no association between birth weight and breast cancer, in contrast to the vast literature [4,6-10]. Also in contrast to our findings, an earlier Danish study by Ahlgren *et al*. based on the same data source that included 106,504 women, but without information on MD, detected a significant, positive association between birth weight and breast cancer [7]. However, this study [7] included younger women and had a 9-year shorter follow-up (until 2000) than ours, resulting in different age distribution of breast cancer cases. In our study, based on screened women older than 50 years, 69.9% and 92.2% of the breast cancer cases were older than age 60 and 55, respectively, whereas Ahlgren *et al*. had 4.1% and 18.0% of cases older than age 60 and 55, respectively. Nonetheless, the age-specific associations agree rather well: for breast cancers older than age 60, Ahlgren *et al*. [7] found a relative risk (RR per kg birth weight) of 0.77 (0.56 to 1.07), whereas we detected HRs of 0.82 (0.66 to 1.02); for breast cancer cases aged 50 to 54 and 55 to 59 years, Ahlgren *et al.* reports RR of 1.08 (0.92 to 1.25) and 1.09 (0.91 to 1.32), respectively, where we, for breast cancer diagnosed between 50 and 59 years, found OR of 1.01 (0.75 to 1.36).

Thus, these two sets of partially overlapping Danish data point at a lack of an association between birth weight and breast cancer from ages 50 to 59 years and a slight indication of a negative association at ages 60 and older. A careful look at the existing literature also supports this notion. Reviews by Ruder *et al*. [6] and Michels and Xue [34] suggest that evidence of a birth-weight effect is mixed and strongest for premenopausal breast cancer [6]. Xu *et al*. [10] showed that the OR from meta-analyses based on studies of premenopausal breast cancer is 1.37 (0.98 to 1.92), whereas postmenopausal is 1.13 (0.85 to 1.51). Indeed, studies with data on both pre- and postmenopausal breast cancer generally find associations with birth weight only for the first group. Oberg *et al*. [35] reports significant and positive associations between birth weight and breast cancer diagnosed before age 50, but inverse nonsignificant associations for cancers after age 50 [35]. Similarly, three studies [36-38] found positive associations between birth weight and premenopausal, and inverse [36,37] or neutral [38] associations with postmenopausal breast cancer.

The mechanisms behind our finding that MD may be a mediator explaining the inverse association between childhood body fatness and breast cancer risk are not well understood. A pathway suggesting direct influence of childhood body fatness on the development of mammary tissue during adolescence [39] is likely, and supported by strong inverse associations with MD observed in current and previous studies [17,18,22-24]. One hypothesis suggests the relevance of sex hormones, higher in girls with more body fat, which are associated with earlier differentiation of breast tissue, resulting in cells less susceptible to malignant transformations [40]. Another theory involves adolescent growth, as childhood body fatness is associated with lower levels of insulin-like growth factor 1 [41,42] and slower adolescent growth, a possible pathway to reduced breast cancer risk [5,6]. In any case, our findings concur with the increasing evidence that the early life exposures and years before first pregnancy, when the mammary glands differentiate and the terminal structure of mammary tissue is determined, are critical in establishing breast cancer risk [5,6].

The current study benefited from a large cohort of women with prospectively collected data on anthropometric childhood factors, MD, and breast cancer, with minimal possibility of recall, information, or selection bias. We detected a strong effect of birth cohort on MD, finding that younger cohorts of women (born in 1945 to 49) had significantly higher MD than women from the oldest cohorts (1930 to 1934), in agreement with Hellman *et al*. [43]. Our study expands on evidence provided by Harris *et al*. [27] about the influence of MD on the association between body size in childhood and breast cancer risk, adding novel results on birth weight and height.

Limitations of this study include the lack of adjustment for other relevant breast cancer covariates at the time of screening, including menopausal status, age at menarche, age at first giving birth, parity, hormone replacement therapy (HRT) use, socioeconomic status, education, physical activity, alcohol use, and others, and possible bias in our estimates due to confounding. However, studies with covariate information available suggest that full adjustment for these enhanced crude associations between birth weight and MD, making them statistically significant [21,23], whereas it did not affect associations of BMI to MD [23,24,27] or of height to MD [23]. Furthermore, we did not have information on BMI at the time of screening, which Lope *et al*. [23] adjusted for, showing no change in estimates of an association between prepubertal weight and MD, as compared with crude estimates. However, adult BMI could be an intermediate variable on the causal pathway between childhood BMI and MD, as pointed by Harris *et al*., who therefore did not to adjust for it in their analyses [27]. Finally, Dorgan *et al*. showed that association of BMI at age 8 to 10 with two different measures of MD was robust to adjustment for adult BMI: effect estimates for percentage dense breast volume were attenuated by a half, but remained inverse and statistically significant, whereas estimates for absolute dense breast volume remained virtually unchanged [24]. Thus, evidence from literature suggests limited possibility of bias in current study due to confounding. Furthermore, adjustment for adult body size for variables, which are on the causal pathway between birth weight/childhood body size and MD/breast cancer risk, is arguably inappropriate and may lead to an artifactual statistical effect [44].

Another limitation is the possibility of BMI tracking, implying that the findings of inverse associations between childhood BMI and MD in adult life would be expected if BMI were tracked through life. As correlations between child and adult BMI strengthen with age, if the observed associations were due to BMI tracking, we would expect the associations of BMI and MD to be much stronger at 13 years of age versus 7 years of age; however, this is not the case here (Table 3). Furthermore, if BMI tracking were to account for the observed associations, then adult BMI should have a stronger association with MD than childhood BMI. Again, this seems not to be the case here, as a related Danish cohort study on adult anthropometry (without data on childhood anthropometry) and MD in 5,937 women reported an inverse association between BMI at ages 50 to 65 and MD. Per SD increase in adult BMI, the odds of having mixed/dense breasts were 0.51 (0.48 to 0.54) (unpublished data); this estimate is similar to the estimates of associations of childhood BMI and MD observed in our study (Table 3). Although unlikely, even if the observed associations between BMI and MD are largely due to tracking, it still leaves open the possibility that the causal processes creating the association between BMI and MD could be operating early in life. If, on the contrary, we assume we had found no association between childhood BMI and MD/breast cancer, whereas one had adult BMI, this would also be very important, because it would indicate that the adult association is based on weight gain in adulthood, and hence the scenario for both exploring the mechanisms and opportunities for prevention would be very different. Finding the associations in childhood that account for the adult association exclude this possibility. Still, the robustness of associations observed in current study to adjustment for other breast cancer risk factors, including BMI to address the possibility of tracking in more detail, will be examined in a subsequent study, in a subset of women from the current data set who participated in Danish Diet, Cancer and Health cohort [45].

The participation rate in the Copenhagen mammography screening program in the period from 1991 to 2001 was between 67% and 70% [46], and women who refused to participate were more likely to be unmarried, older, of non-Danish origin, and have less contact with health care (primary physician or dentist), than did screened women [47]. However, a U-shaped curve was found for an association between education and screening nonparticipation, reflecting high rates of nonparticipation among both, women with highest and lowest education [48].

We used a dichotomized outcome of high (mixed/dense breasts) and low (fatty breasts) MD, as no other measure of MD was available. In contrast, a wide variety of measures of MD were used in related studies, including the Boyd semiquantitative scale with six levels (A to F) [18,23], Wolfe score of four qualitative categories [17,21], BI-RADS [25], PMD [19,20,22,26,27], or absolute and percentage dense breast volume [24]. However, the dichotomous outcome has been used successfully earlier in a study of MD and breast cancer mortality [32], and showed an expected doubling of the breast cancer risk in women with mixed/dense compared with women with fatty breasts, with HR or 2.34 (1.97 to 2.78) (Table 3), in agreement with Boyd *et al*. [2]. Furthermore, we successfully validated dichotomous MD measure in a subset of 118 women from this study who had their negative screening mammograms reevaluated and assigned BI-RADS, for a related study [49]. Specifically, in these 118 women, we compared dichotomous MD outcome (fatty, which should be equivalent to BI-RADS code 1 and part of 2; and mixed/dense, which should be equivalent to part of BI-RADS code 2, 3, or 4) with BI-RADS code, and found rather good agreement: among the 31 women coded as having fatty breasts, 32% were estimated as having BI-RADS code 1, 61% BI-RADS code 2, and 7% BI-RADS code 3, whereas among 87 women with mixed/fatty breasts, 1% had BI-RADS code 1, 31% BI-RADS code 2, 62% BI-RADS code 3, and 6% BI-RADS code 4 at reevaluation.

## Conclusions

Our study indicates that birth weight is not related to MD or breast cancer risk after age 50 years. High BMI in girls is a strong determinant of a favorable MD, whereas height is not related to MD. Childhood body fatness is inversely associated with the breast cancer risk, possibly via a mechanism mediated by MD, at least in part, whereas childhood tallness is positively associated with the breast cancer risk, however, via a pathway that seems independent of MD.

## Abbreviations

BMI: Body mass index; CI: confidence intervals; CPR: unique personal identification (number); CSHRR: Copenhagen School Health Records Register; HR: hazard ratio; HRT: hormone replacement therapy; MD: mammographic density; MPD: mammographic percentage density; OR: odds ratio.

## Competing interests

The authors declare that they have no competing interests.

## Authors’ contributions

All authors made substantial contributions to conception and design, analysis, and interpretation of data, and critical review of the manuscript. ZJA carried out the design of the study, performed the statistical analyses, and wrote the manuscript. JLB secured funding, assisted in designing the study, interpretation of the results, and the revision of the manuscript. KB coordinated the data acquisition and assisted with the statistical analyses. IV made mammographic measurements, helped to draft the manuscript, and assisted in interpretation of data. TIAS participated in the acquisition of data, helped with the design of the study, interpretation of the results, and the revision of the manuscript. EL conceived of the study and participated in the designing of the study and the interpretation of the results. All authors participated in the drafting and revision of the manuscript. All authors read and approved the final manuscript.
